# Knowledge, skills and competency retention among health workers one year after completing helping babies breathe training in South Sudan

**DOI:** 10.11604/pamj.2019.33.175.17560

**Published:** 2019-07-05

**Authors:** Christopher Vunni Draiko, Khemika Yamarat, Alessio Panza, Judith Draleru

**Affiliations:** 1 Chulalongkorn University, Bangkok, Thailand; 2 Juba Teaching Hospital, Juba, Sudan

**Keywords:** Knowledge, skills, competency, retention, neonatal resuscitation, education

## Abstract

**Introduction:**

This study aimed to evaluate the long-term retention of knowledge, skills, and competency of health workers who completed a Helping Babies Breathe (HBB) training program and its effect on newborn mortality.

**Methods:**

A quasi-experimental pre- and post-intervention study was conducted. Participants were health workers selected based on their previous training on HBB protocols. Participants were assessed for knowledge, skills, and competency in March 2017 (immediately before and after training), June 2017 (three months after training), and September 2018 (one year after training). Assessments were conducted using HBB questionnaires, checklists and practical skill drills. Mean scores were obtained and ANOVA, chi-squared test, and Pearson's test were used for pre intervention, post intervention, and one-year-after comparisons. The effect of training on the management of newborn asphyxia was assessed based on a review of the delivery registry at a maternity and children's ward. The scores were group into percentages and averages means and were computed using chi-squared tests.

**Results:**

Despite improvements in knowledge, skills, and competency three months after training, participants showed a marked decline one year after training. Knowledge increased from 42.5% pretest to 97% posttest but decreased to 84.5% three months' post training and further decreased to 69.4% one year post training. Skills increased from 26.1% pretest to 94.4% posttest, remained at 94.4% at three months, and decreased to 77.0% at one year. Simple resuscitation scores increased from 26.9% to 88.8% pre- and posttest, remained roughly at three months and decreased to 76.4% at one year. For complex resuscitation, scores decreased from 90.9% posttest to 76.9% at one year. The assessments at one year indicated a need for support and practice, especially with bag-mask ventilation.

**Conclusion:**

The immediate evaluation of health workers after HBB training showed significant increases in knowledge, skills and competency in neonatal resuscitation. However, this declined after one year. The training also resulted in decreased neonatal mortality.

## Introduction

Of the 200,000 children born annually in South Sudan, an estimated 40% die in the first month of life [1]. Newborn mortality accounts for 39% of all under-five deaths in South Sudan. Over the last decade, the decline in the newborn mortality rate in South Sudan has been slower than the global average [2]. An estimated 136 million infants are born each year and this figure is expected to rise in the coming years. The highest risk of newborn mortality is present during the first day of life. Death during this period accounts for almost 5% of newborn deaths worldwide [3, 4]. Globally and in South Sudan, the main direct causes of newborn deaths are usually infection-related complications (26%); intrapartum complications (24%), including birth asphyxia; preterm delivery (34%) and congenital abnormalities (9%). In South Sudan, 20% of newborn deaths are associated with birth asphyxia [5, 6]. The transition from intrauterine to extra uterine life requires the initiation of breathing, which is a critical physiological change required for newborn survival. Research has indicated that most newborns initiate breathing within 30 minutes, and an estimated 10% breathe when they receive drying and stimulation from health workers. Three percent of newborns require positive pressure (PPV) while another 2% need ventilation and intubation [7, 8]. Training health workers on effective and timely newborn resuscitation could reduce newborn asphyxia and improve survival rates. To address inadequate resuscitation training in health facilities in low-resource areas and improve neonatal outcomes, in 2010 the American Academy of Pediatrics developed a newborn-care training program for healthcare professionals called Helping Babies Breathe (HBB) [9]. The HBB curriculum is designed to train birth attendants in low-resource countries in neonatal resuscitation. It is evidence based and specifically geared toward reducing global neonatal mortality. HBB focuses on the essential steps of resuscitation, including the birth evaluation of the infant, breathing stimulation, and ventilation during the critical “golden minute” after birth [10]. The HBB curriculum promotes active learning and hands-on practice using newborn simulators, self-reflection, and group discussion and feedback after completing a task. It also focuses on paired learning, which is tested based on four formative assessments. Competence and performance can also be determined based on multiple-choice questionnaires, bag-mask ventilation (BMV) tests, and objective structural clinical examination consisting of sections A and B (OSCEA&B). Before its global launch, HBB curriculum assessment was modified based on the study of Singhal *et al.* on the educational evaluation of HBB assessment in Kenya and Pakistan. Further studies of HBB curriculum assessment in Africa and Asia using pre- and posttest scores showed significant gains in resuscitation knowledge and skills immediately after training [11, 12]. However, those studies did not conduct detailed examinations of learners' qualifications and the characteristics of their performance. Understanding learners' performance and competence could help refine the HBB curriculum to promote the retention of neonatal resuscitation knowledge and skills tailored to specific learners. Studies on the effectiveness of newborn resuscitation training programs have shown increased knowledge, skills, and competency after training, which was sometimes retained for one year. The evaluation of newborn resuscitation training has shown immediate increases in knowledge, practical skills, and competency, with improved newborn outcomes [13]. Nevertheless, there has been limited rigorous evaluation of the retention of knowledge, skills, and competency, and of subsequent newborn outcomes, in low-resource and post conflict settings, such as South Sudan. Training outcomes depend not only on the extent to which such knowledge and skills are retained but also on the ability to apply them at appropriate times. This depends on several factors, including conditions in the clinical environment, regular supervision, settings, practice opportunities, and the availability of supplies and appropriate equipment [14]. Providing appropriate training, offering refresher training and support, and ensuring good training environments facilitate long-term knowledge, skills, and competency retention among health workers in low-resource settings [14]. In South Sudan, nurses and midwives typically manage normal deliveries, and birth asphyxia is usually not recognized early enough. Due to critical shortages, medical doctors are usually not involved until the late stages of managing birth asphyxia, even in major hospital settings. The present study aimed to evaluate the retention of knowledge, skills, and competency among health workers and the effects on newborn mortality one year after implementation.

## Methods

**Trial background:** the trial in this study was registered with the Pan African Clinical Registry [15]. The study trend statement is available as a supporting document. The study originally aimed to measure only improvements in health workers' knowledge, skills and competency. The measurement of newborn mortality was conceived later, leading to late registration.

**Sample size:** sample-size calculation was based on the ability to detect a 20% increase in knowledge, practical skill, and competency, as well as a 20% reduction in newborn mortality, with an error of 0.05, 20% and a dropout rate of 50%. Using G*Power v. 3.1, we determined a sample size of 74 participants in each group to account for losses. However, due to the ongoing conflict in South Sudan, the actual participants for both groups were less than the estimated sample size.

**Selection criteria:** the selection criteria were as follows: medical officers/doctors, nurses, midwives, maternal child health officers, community health workers, and clinical officers working and practicing in maternities, operating theaters, or children's wards; health providers self-reporting that they provide routine care services in delivery and neonatal units or departments and health workers willing to be available for data collection during the period of study.

**Recruitment setting:** health workers were identified and recruited from maternity wards, newborn operating theaters, and children's wards at Juba Training Hospital (intervention site) and Wau Teaching Hospital (control site). After completing the recruitment process, invitations were sent to those who met the criteria to participate in the study. Written informed consent was obtained from the participants. All newborns delivered in maternities, newborn units and operating theatres who met the inclusion criteria were included in the study.

**Location of data collection:** data on health workers' pre- and post-training knowledge and practical skills and the records of newborn asphyxia and deaths were collected for both the intervention and control groups from the maternity wards, operating theatres, and newborn units in the areas of practice. Retention evaluation was conducted at Juba Teaching Hospital in the Republic of South Sudan. The pre- and post-training assessment was completed in March 2017, and a three-month assessment of knowledge, skills and competency retention and neonatal mortality was performed in June 2017. The one-year assessment was conducted in September 2018. During the evaluations, health workers who were available and had received previous HBB training in both hospitals were approached and interviewed according to the established exclusion and inclusion criteria. The objective of this study was to assess changes in health workers' knowledge, psychomotor skills, and competency regarding managing neonates with birth asphyxia after receiving training, as well as the changes in perinatal mortality rates due to asphyxia within 24 hours of birth. We hypothesized that HBB training would result in a 20% increase in knowledge and skills, and a corresponding reduction in mortality among newborns with asphyxia. The primary expected outcome was an improvement in health workers' knowledge, skills, and competency after HBB training. The secondary expected outcome was a reduction in mortality among newborns with asphyxia in the first 24 hours after birth. The study design was approved by the South Sudan Ethical Committee, Ministry of Health. It was also reviewed by Chulalongkorn University, College of Public Health Sciences, as part of a PhD project. Informed consent was sought from each health worker available at the time of evaluation.

The training was led by the lead researcher, who was trained in Liberia on HBB. In December 2016, four midwives completed the HBB “trainers of trainer's” (TOT) workshop and became research assistants and facilitators. The first part of the TOT workshop covered teaching methodology, neonatal resuscitation using the HBB model, skills evaluation and practical training. The second part of TOT involved HBB simulation drills, birth preparation, routine newborn care, “golden minute” training and newborn ventilation. The last part of the TOT program involved facilitation and coaching skills using the American Pediatric Association's HBB model. The four midwives supervised and remain at the delivery sites throughout the period of the study. After TOT completion, health workers were recruited for the intervention (Juba) and control (Wau) groups. All health workers working in maternities, neonatal wards and antenatal clinics were approached to participate in the study. The study was explained to the health workers in Arabic and English, and they signed informed consent forms prior to participation. Pre training assessment was conducted for demographics, previous knowledge and exposure to HBB training. Also, birth registries were reviewed for the number of births, neonatal asphyxia and mortality in the previous year. A multiple-choice questionnaire, a simulated environment using a bag-and-mask checklist for psychomotor skills and OSCEA&B were used to assess competency immediately pre- and post-intervention, at three months and at one year.

The initial training program covered preparation for birth, routine care, the golden minute and newborn BMV. Individual training involved simulation drills, performance feedback and lectures by facilitators. Participants were allowed to practice several drills and demonstrations using mannequins. Demonstrations were accompanied by explanations of tasks related to helping newborns breathe. In each training session, health workers were asked to evaluate the training using five-point Likert scales. In the HBB training, trainers and trainees reviewed training modules and conducted practical sessions on newborn asphyxia, routine care and ventilation. Participants practiced on mannequins and were provided with the proper equipment; their performance was rated by the facilitators. The mannequins, representing a mother and her newborn, were placed on the resuscitation table. Each situation presenting the condition of the baby and mother was controlled, with the participants providing the appropriate care. The raters ensured that the participants completed the skill-based checklist to assess their performance during the practical drills. Participants performed the drills in either Arabic or English, which are commonly spoken in South Sudan. The tasks performed by the participants were explained to the midwife rater as the participants conducted the resuscitation drills.

Raters assigned a 1 when the task was correctly completed and a 0 otherwise. Health workers needed to obtain 80% or above in all three aspects of knowledge, skills and competency to be considered competent to help newborns breathe. Post training and intervention assessment were done immediately and at three months using the HBB knowledge, skills and competency checklist. Assessment were categorized into knowledge, practical skills and competency. The sum of the scores was calculated by determining the degree of completion of each task in knowledge, skills and competency. The total score for each task was 80% for knowledge, skills and competency. All participants who completed the initial HBB training and were present one year later were asked to participate in the HBB assessments to determine their long-term knowledge, skills and competency retention. The one-year assessment used the same checklist that was used in the initial training. In the one-year evaluation, each participant was assigned a knowledge, skills and competency score presented as the percentage of correctly performed items. Participants were asked to complete the same five-point Likert scale survey and express their confidence in HBB practice. The mean scores and significance levels within and between the groups were tested using repeated ANOVA and chi-squared tests in the three performance areas of knowledge, skills and competency. Newborn mortality was determined and tested with Pearson's chi-squared test. Statistical analyses were performed using SPSS v. 20 and p<0.05 was considered statistically significant.

**Assessment for simple newborn resuscitation:** all participants in the intervention group were assessed for simple resuscitation using Objective Structural Clinical Examination (OSCE A). The tool is made up of 13 observation steps consisting of scripted information on preparation for birth, drying the baby thoroughly, ability to recognize the baby is not breathing, positioning of the head and clearing the airway, evaluation of the breathing, clamping or tying and cutting the cord, position skin to skin and communication with mother. Successful completion by the participants requires a total score of 10 correct of 13 steps which must include: dries the newborn thoroughly, recognizes baby is not crying and positioning and clearing the airway. OSCE A is a performance assessment of preparation for birth and routine newborn care, and a learner must perform = 80% (10 of 13 steps) correctly to pass, including three essential steps. OSCE A is a performance assessment of preparation for birth and routine newborn care and a learner must perform 80% (10 of 13 steps) correctly to pass, in addition to three essential steps for newborn survival.

**Assessment for complex newborn resuscitation:** similarly, participants were assessed for complex neonatal resuscitation using Objective Structural Clinical Examination (OSCE B). The tool is made up of 18 scripted scenarios on preparation for birth, drying of the baby, recognizing the baby not breathing, ventilation at 40 breathes per minutes (30-50), looking for chest movement, evaluating breathing, calling for help, improving ventilation thorough, repositioning the head, reapplication of mask, clear secretion, opening mouth slightly and squeezing the bulb hardly. The 18 items reflect the key components of the training course for newborn survival. Each item was scored 1 if carried out correctly and any partial or incorrect action were scored zero. Similar to OSCE A, a learner must perform 14 of the 18 steps correctly to be evaluated to have the competency to help newborn with asphyxia to breathe.

**Daily bag and mask drills assessment evaluation:** participants in the intervention group performed regular equipment checks and run drills on NeoNatalie using a checklist. In situations, where participants encounter neonates with birth asphyxia, the supervisor conducts group evaluation and allows participants evaluate their performance using the self-evaluation checklist. All the participants who completed resuscitation procedures signs in the delivery registers indicating the procedures undertaken and neonatal conditions.

**Hospital registry and forms:** data on neonatal mortality were collected from the hospital register at the delivery room, operating theater and neonatal unit admission book pre- and post-implementation for June 2017-June 2018. Record were collected on number of deliveries, deliveries, neonates with breathing problems; neonates resuscitated using HBB protocols and the perinatal mortality due to asphyxia outcome immediately post-training and end of intervention. The record/data collected pre, post and I year evaluation was used determine the outcome/changes of the intervention within the period. Cases of pre maturity were handled carefully and death due to asphyxia was only recorded determined after experienced neonatal doctor /midwife reviewing the case note indicating the cause of death being asphyxia rather than pre-maturity and it complications.

## Results

**Figure 1:**
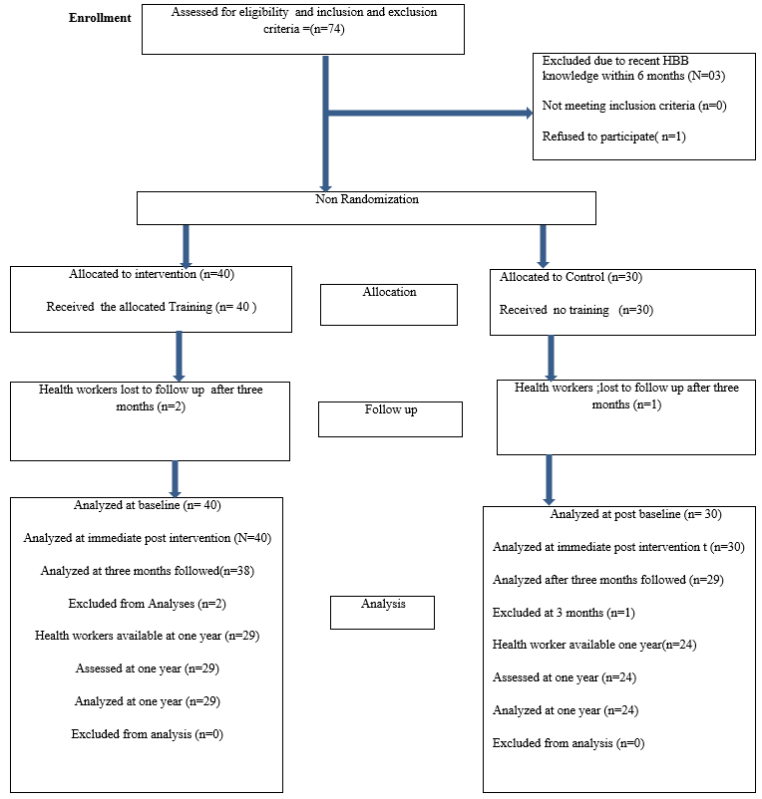
Flow chart for recruitment and allocation health workers

**Characteristics of health workers:** seventy health workers completed the pre training course and 67 were evaluated at three months. Fifty-three participants who completed the original training and were evaluated at three months were available for the one-year evaluation. Figure 1 shows the flowchart for the recruitment of health workers.

**Characteristics of health workers:** a total of 58.6% of the health workers in the intervention and 66.7% in the control group worked full time in maternity wards. Participants attended an average of 115 deliveries and managed 7.7 asphyxia cases per year. Table 1 shows the demographic characteristics of the health workers one year after implementation.

**Table 1:** Demographic characteristics

		Baseline							One year			
		Intervention		Control					Intervention		Control	
Demographic Characteristics		Freq. (N=40)	(%)	Freq. (N=30)	(%)		p-value	Statistical test	Freq. (N=29)	(%)	Freq. (N=24)	(%)
**Age in years**	25-35	25	62.5	20	66.7	0.130	0.719	Chi-squared	17	58.6	15	62.5
	36 above	15	37.5	10	33.3				12	41.4	9	37.5
	**Total**	**40**		**30**					**29**		**24**	
**Gender**	Male	7	17.5	6	20.0	0.071	0.790	Chi-squared	4	13.8	3	12.5
	Female	33	82.5	24	80.0				25	86.2	21	87.5
	**Total**	**40**		**30**					**29**		**24**	
**Education level**	Primary eight	5	15.0	4	13.3	0.748	0.781	Fisher Exact	4	13.3	3	12.5
	Secondary	2	7.5	4	13.3				3	10	4	16.7
	College/tertiary	31	77.5	22	73.3				19	63.3	17	70.8
	Diploma in Midwifery	1	2.5	0	0				1	3.3	0	0
	Community HW Training	1	2.5	0	0				2	7.0	0	0
	**Total**	**40**		**30**					**29**		**24**	
**Professional qualification**	Nurse	12	30.0	10	33.3	5.690	0.623	Fisher Exact	9	31.0	10	41.7
	Midwives	17	42.5	11	36.7				10	34.5	9	37.5
	Maternal child health officer	3	7.5	1	3.3				3	10.3	0	0.0
	Nurse practitioner	1	2.5	0	0				1	3.4	0	1.0
	Clinical officer	2	5.0	2	6.7			Fisher Exact	2	6.9	2	8.3
	Community health workers	4	10.0	2	6.7				4	13.8	2	8.3
	Skilled birth attendants	1	2.5	3	10.0				0	0.0	0	0.0
	Intern doctor			1	3.3				0	0.0	1	4.2
	**Total**	**40**		**30**					**29**		**24**	
**Primary area**	Newborn care	11	27.5	8	26.7	5.987	0.097		8	27.5	11	45.8
	Sick children ward	4	10.0	1	3.3				3	10.3	2	8.3
	Maternal and newborn care	25	62.5	17	56.7				18	62.1	10	41.6
	Obstetrics/obstetrician	0	0	4	13.3				0	0	1	4.1
	**Total**	**40**		**30**					**29**		**24**	
**Current place of work**	Maternity ward	23	57.5	19	63.3	4.135	0.129	Fisher Exact	17	58.6	16	66.7
	Children’s ward	16	40.0	7	23.3				11	37.9	4	16.7
	Operating theater (OT)	1	2.5	4	13.3				1	3.4	4	16.7
	**Total**	**40**		**30**					**29**		**24**	
**Monthly income**	300-1000 SSP	18	45.0	8	26.7	2.896	0.235	Chi-square	10	34.5	7	29.2
	1001-2,000 SSP	14	35.0	16	53.3				11	37.9	12	50.0
	2,001 SSP and above	8	20.0	6	20.0				8	28	5	21
	**Total**	**40**		**30**					**29**		**24**	
**Duration of practice**	≤ 1 year	12	30.0	6	20.0	0.980	0.806		7	24.1	4	16.7
	Two–three years	10	25.0	8	26.7				7	24.1	7	29.2
	Four–five years	5	12.5	5	16.7				5	17.2	4	16.7
	Over five years	13	32.5	11	36.7				10	34.5	9	37.5
	**Total**	**40**		**30**					**29**		**24**	

**Knowledge retention:** the mean scores for the participants at pretest, posttest, three months and one year, tested by repeated-measures ANOVA, showed significant increases in knowledge between the pretest and the immediate post intervention (mean difference increase of 55.2 (50.9-59.6; p<0.05). This decreased slightly at the three-months follow-up, with a mean difference of 13.3 (-17.7–8.87; p<0.05). At one year, the mean score further decreased, from 84% to 69.4%, in the 17 domains that were assessed, with a mean difference of -15.0 (-22.7-7.4). The changes in scores at one year were statistically significant. Meanwhile, the assessment established that knowledge scores increased for health workers in the control group from 50% to 61%, with a mean difference of 10.5 (0.54-20.6). The changes in knowledge among the control group were significant (Table 2).

**Table 2:** HBB knowledge, psychomotor skills, and competency one year after post training

	Intervention		*P*-value^a^	Control		*P*-Value
Variables	Mean	*Mean df. (CI)*		Mean	*Mean df. (CI)*	
**HBB Knowledge**						
**Pretest**	42.5± 17.3			48.0±13.9		
**Posttest**	97.8±3.4	55.2(50.9-59.6)	<0.001	51.2±11.2	3.1(-3.0-9.4)	0.9
**3-month follow-up**	84.5±7.4	-13.3(-17.7-8.87)	<0.001	50.6±16.9	-0.3(-0.1-6.0	0.9
**1-year follow-up**	69.4±18.8	-15.0(-22.7 - 7.4)	<0.001	61.2±20.3	10.5(0.54-20.6)	0.04
**HBB Psychomotor skills (Bag and Mask)**						
**Pretest**	26.1±19.9			*	-	-
**Posttest**	94.4±8.5	69.2(62.8-75.7)	<0.001	43.8±16.7		
**3-month follow-up**	94.5±8.2	0.1(-03-08)	<0.001	40.3±20.5	3.4(11.0-4.10)	0.37
**1-year follow-up**	77.0±21.8	-17.5(-27.2- -7.8)	0.001	56.5±25.5	16.2(1.2-31.2)	0.04
**HBB competency for simple neonatal resuscitation (OSCE A)**						
**Pretest**	26.9±14.6			*	-	-
**Posttest**	88.8±8.5	61.2(57.0-66.7)	<0.001	38.9±8.5	-	-
**3-month follow-up**	88.6±8.6	03.1(-4.6-532)	0.9	38.0±9.1	2.65(-8.42-3.1)	0.36
**1-year follow-up**	76.4±13.6	-12.2(-18.3- -6.1)	<0.001	53.9±11.8	15.1(8.7-21.4)	<0.001
**HBB competency for complex neonatal resuscitation (OSCE B)**						
**Pretest**	17.5±8.9			**	-	-
**Posttest**	90.9±7.1	73.47(69.5-77.36)	<0.001	36.5±13.0	-	-
**3-month follow-up**	88.3±10.8	-274(6.71-1.22	0.17	33.1±8.7	-3.63(-8.2-0.93)	0.12
**1-year follow-up**	76.9±11.6	-13.6(-19.8- -7.4	<0.001	53.4±21.7	21.0(10.9-31.0)	<0.001

Scores expressed as mean difference.

* Significance level at 0.005 post intervention, 3-month follow-up, and one year. P-value within intervention group tested by repeated ANOVA, 0.001, and 0.001 between immediate post intervention and 3-month follow-up.

** No baseline conducted for control group for bag and mask, OSCEA&B.

**Skills retention:** skills retention among the participants was assessed using seven domains at pretest, posttest, three months and one year. One-year mean scores declined from 94.5% to 77% from the three-month assessment. The decline was evident across all seven domains, with mean a difference of -17.5 (-27.2-7.8). The results showed a marked increase in mean scores among the control group (Table 2); the mean skill score increased from 40.3% to 56.5%. The mean difference in skills was 16.2 (1.2-31.2), which was statistically significant (0.04).

**Simple resuscitation competency:** simple resuscitation competency declined among the intervention group and greatly increased in the control group at one year compared to the assessment at three months. There was a significant decrease in competency between the three months and one year of training: 88.6±8.6 to 76.4±13.6 (mean difference: -12.2 (-18.3-6.1); p<0.001). The mean decrease between three months and one year was significant. Competency retention in the control group showed a significant increase from 38.0±9.1 to 53.9±11.8 (mean difference: 15.1 (8.7-21.4); p< 0.001) (Table 2).

**Complex resuscitation competency:** complex resuscitation competency after one year showed a marked decreased in the intervention group and a significant increase in the control group. In the intervention group, the mean competency score decreased from 90.4±8.6 to 76.9±11.6 (mean difference: -13.6 (-19.8-7.4); p<0.001). In the control group, it increased from 32.4±8.3 to 53.4±21.7 (mean difference: 21.0 (10.9–31.0); p<0.01).

**Figure 2:**
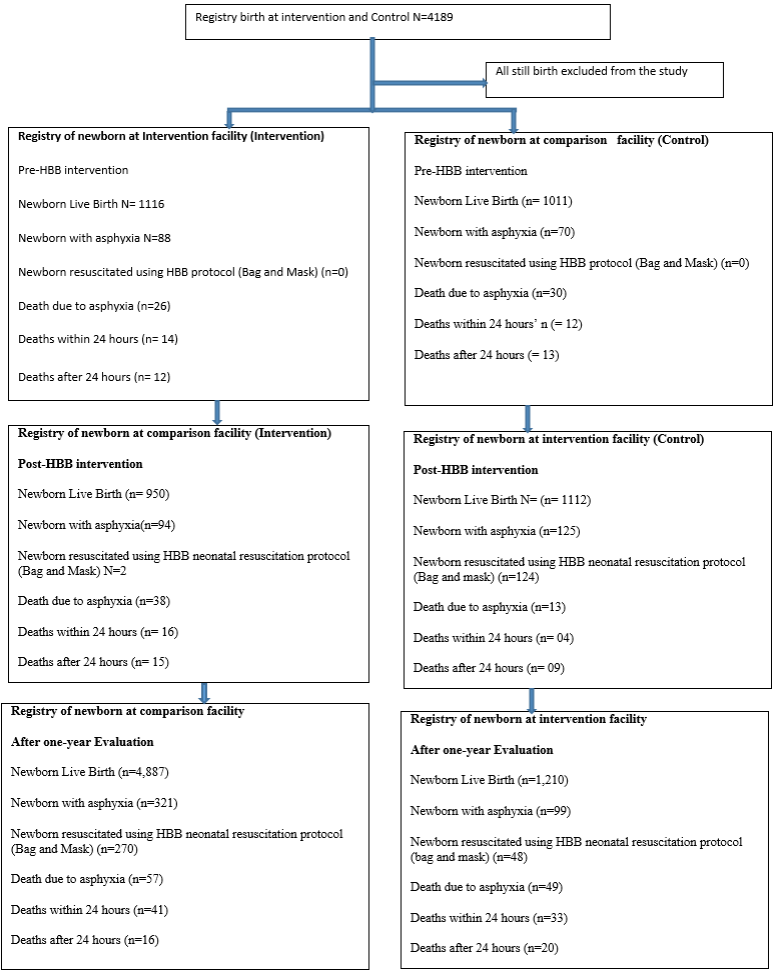
flowchart for birth registries pre- and post-implementation

**Early newborn mortality:** a review of the records from June 2017 to June 2018 found that 6,097 live births were registered in the studied maternity, newborn and antenatal wards (4,887 in the intervention hospital and 1,210 in the control hospital). All births were assisted by health workers trained in HBB. Figure 2 shows the flowchart for birth registries pre- and post-implementation.

**Newborn mortality:** the one-year evaluation showed a significant increase in newborns with asphyxia being resuscitated in both hospitals. More newborns in the intervention hospital received neonatal support (86.7%) compared to the control hospital. In the intervention hospital, mortality due to asphyxia decreased from 30.7% (p = 0.001) to 17.9% (p = 0.001), which is significant. There was also a significant reduction of early newborn mortality in the control hospital, from 69.2% (p = 0.110) to 49.4% (p = 0.001). The results indicated that more newborn deaths occurred within 24 hours of birth in both hospitals (Table 3).

**Table 3:** Early newborn mortality due to asphyxia one year after helping babies breathe (HBB) training

Variable		Before Intervention		After Intervention			One year post intervention		
	Intervention/Control	Freq	Percent(%)	Freq	Percent(%)	P. Value	Freq.	Percent (%)	P.Value^a^
Total live births	Intervention	1116	52.40	1112	53.9	-	4,887	80.2	0.000
	Control	1011	47.50	950	46	-	1,210	19.8	0.000*
Newborn Birth Asphyxia	Intervention	88	55.7	125	57.1	0.18	312	75.9	<0.001*
	Control	70	44.3	94	42.9	-	99	24.1	<0.001*
Newborn resuscitated using HBB	Intervention	0	00	124	98.4	0.001*	270	86.7	0.001*
	Control	0	00	2	1.6	0.114	48	48.4	0.001*
Asphyxia deaths	Intervention	26	50.9	4	30.7	0.001*	57	17.9	0.001*
	Control	25	49	9	69.2	0.110	49	49.4	0.001*
Death within 24 hours	Intervention	14	51.9	4	23.5	0.001*	41	71.9	0.001*
	Control	13	48.1	13	48.1	0.110	33	67.3	0.001*
Death after 24 hours	Intervention	12	50.0	9	33.3	0.000*	16	28.0	0.001*
	Control	12	50.0	18	66.7	0.112	20	32.7	0.001

Significance level at 0.05. Rounded at 1 decimal place; tested by Fisher’s exact test 2x2 sided significance for birth asphyxia, resuscitated using HBB, newborn death within and after 24 hours.

## Discussion

This study aimed to evaluate knowledge, skills and competency retention as well as the effect of HBB training on newborn mortality at a public tertiary hospital in South Sudan. To our knowledge, this is the first rigorous study of HBB conducted in South Sudan The knowledge, skills and competency of the intervention and control participants were evaluated one-year post training and compared to the status at three months.

### Participants' HBB knowledge

The mean knowledge passed rates using the HBB multiple-choice questionnaire were 42.5% and 48% for the intervention and control groups, respectively. There were no significant differences between the intervention and control groups during the baseline assessment, though the control group scored slightly higher. No known formal psychometrics have been performed beyond field tests on HBB multiple-choice questions. Despite differences in the participants' education and professional characteristics, HBB was beneficial and appropriate, regardless of profession, as demonstrated by the statistically significant gains in posttest scores.

Importantly, the health workers who received training were able to achieve high scores in simulated environments despite a lack of previous HBB training. Furthermore, the results showed that helping babies is an effective way to address a lack of knowledge among the health workers who are often the first to act in resuscitating newborns with asphyxia. A study of HBB implementation in Honduras obtained a mean baseline knowledge score of 46%, which is close to our results for both the intervention (42.5%) and control groups (48%) [16]. The same study found that health workers with prior training scored higher at 69%. This is different from our results for the control group, where most had been exposed to resuscitation training but had lower scores. In India, health workers receiving HBB training for the first time did not score higher than those who had received it previously. Similarly, our study obtained low baseline mean knowledge scores for first-timers, raising concerns about the retention of HBB resuscitation knowledge [16]. The level of knowledge attained after training declined at three months and further declined after one year. In an evaluation of HBB education in Kenya, the pass rate for knowledge increased among trainers (nurses and doctors) from 75% to 95% after a similar training intervention. In the same training, the pass rate for the simulated practical skills and competency was 20% for the health workers (learners) [11,14]. Using a simulated learning environment greatly improved the participants' knowledge of neonatal resuscitation. However, that knowledge was not sufficiently retained after one year. The creators of HBB training noted that long-term knowledge retention posed a significant challenge. Most prior studies have found a similar rapid deterioration in acquired skills and, to a lesser extent, knowledge in the months following the training [17].

### HBB psychomotor skills and competency

In most prior studies of HBB training and evaluation, baseline BMV scores were not obtained [18]. The present study established a baseline level for psychomotor skills and competencies for the intervention before conducting the training. During the study, we administered the BMV OSCEA&B to the intervention group at baseline, immediate intervention and three months. For the control group, it was administered at immediate intervention, three months and one year. In most cases, OSCEA&B was considered too difficult to be administered to the participants during the pre-training period. Based on the pre assessment of the practical skills and competencies of health workers in the intervention group, we tailored the support to each participant's ability and understanding, which facilitated good post training results. This study also found improvements in practical skills and competency after training. Though the skills remained high at three months, there was a significant decrease after one year. Despite this decrease, there was a significant reduction in neonatal mortality. It was surprising to find that health workers retained skills and competency after three months. Similar studies of skills and competency retention in Rwanda and Kenya found that 3-6 months after intervention was the most difficult period for retaining such skills and competency [17].

Another study evaluating a performance checklist to assess neonatal resuscitation skills found that, despite significant improvements in overall test scores and pass rates after training, scores and improvements were disproportionate for those assessments that involved the demonstration of skills (BMV, OSCE A, OSCE B). Miller found that knowledge acquisition occurs earlier and more easily than skill acquisition. In Miller's framework, thinking moves progressively from knowledge (“knows”) to skill demonstration (“knows how”) to performance assessment (“shows how”). In our study, participants demonstrated mastery of neonatal resuscitation knowledge (“knows”), as evidenced by high posttest scores and pass rates on the multiple-choice questionnaire. However, they struggled more with assessments requiring skills and higher-level performance, as evidenced by the low baselines scores for both groups. At one year, however, the practical scores followed Miller's model of knowledge acquisition. The high scores obtained by participants at three months differed from the results of other studies on skill and competency retention. Our study, however, found that practical skills were retained beyond three months. This confirms that HBB is a practical course that requires periodic skill reinforcement through review, problem solving, and self-assessment to ensure high knowledge and skill retention. A study in Ghana assessing neonatal resuscitation training among midwives found that knowledge and skills remained stable within a period of 9-12 months' post training. This largely concurs with our findings regarding practical skill and competency retention. Many similar studies of health workers have highlighted the need for refresher training courses between posttraining and implementation for the retention of knowledge, skills and competencies.

### Early neonatal mortality (ENM) within 24 hours

The training intervention in this study had an impact on newborn outcomes. The data indicated that, prior to the training, most newborns had not received resuscitation following HBB protocols. Health workers were not following the correct steps for resuscitating newborns with asphyxia, including those who required stimulation or BMV for neonates with breathing problems. Additionally, the health workers had previously had difficulty properly identifying newborns with breathing problems and initiating resuscitation within the “golden minute”. However, after HBB training and implementation, the participants' resuscitation skills increased twofold and such skills had been retained at the three-month follow-up.

This study also found a trend toward an overall reduction in newborn mortality within 24 hours three months after implementation at both sites. At the intervention site, newborn mortality within 24 hours decreased by half after the administration of resuscitation by the trained participants. Meanwhile, there was no significant decrease in ENM at the control site. Despite the notable decrease in early newborn deaths at the intervention site, the short period between the baseline and implementation was not sufficient for the results to be conclusive and generalizable. Thus, there is a need for a longer period between baseline and implementation given the variations in newborn registry data, especially in political conflict areas. Nevertheless, this study has shown that training health workers has an effect on their knowledge and practical skills, in addition to reducing newborn mortality.

Few existing studies have investigated the long-term effects of HBB training on early neonatal outcomes. One large before-and-after study of eight hospitals in Tanzania found that HBB training and implementation was associated with a significant reduction in ENM, fresh stillbirth rates and early perinatal mortality [19]. In that study, ENM was reduced from 13.4 to 7.1 per 1,000 live births, and the reduction in ENM was significant for both normal and low birth weights, as well as term and preterm infants. The only difference from our study is that we excluded stillbirth and considered neonates identified as having breathing problems at birth.

A strength of this study is that most participants were nurses and midwives who had had training on newborns and had worked full time in maternity and children wards. Thus, they represented the population that typically encounters and manages birth asphyxia in the hospital setting. Documenting knowledge, skills, competency and birth asphyxia during pre-training, immediate post training, at three months, and after one year provided an opportunity to evaluate the same health workers available at the hospital at the time of assessment. A limitation of this study is that there still exists a large gap in the documentation of medical records and data use among health workers, clerks and managers in hospitals and health facilities in South Sudan. Moreover, this study was limited by excluding stillbirths and premature newborns from the analysis.

## Conclusion

In this study, HBB training significantly improved health workers' knowledge, skills and competency regarding neonatal resuscitation and neonatal mortality rates improved as well. Given the ongoing conflict, we suspected that such training might not have the hypothesized effect, but this was proven wrong. Knowledge, skills and competency were found to decline after one year. This finding concurs with many similar studies conducted in other low-resource countries. HBB implementation positively affected neonatal survival, with a significant reduction in deaths from asphyxia-related illnesses compared to pre implementation rates. HBB training requires the minimally necessary equipment for newborn resuscitation. Periodic refresher training can help increase knowledge, skills, and competency, reduce asphyxia-related deaths and prepare staff for asphyxia-related emergencies. Further quality improvements and refresher training can minimize the deterioration of knowledge, skills and competency. Given the outcomes observed in this study, South Sudan would benefit from scaling up HBB training. The findings indicate that even in conflict settings, health workers can easily acquire knowledge and retain skills. Although skills and competency can be difficult to acquire, the careful selection of teaching methodology and a focus on the practical aspects of the course can improve all aspects of knowledge, skill and competency acquisition. Before HBB training, BMV was challenging for the health workers and often ignored. Identifying the right skills to teach to participants can be difficult. Awareness of the problem areas identified in the pre training helped us determine the best educational approach, focusing on BMV administration within the critical time frame and taking into account the participants' varied training backgrounds and clinical exposure to resuscitation education. Several HBB follow-up studies have raised concerns about declines in basic neonatal resuscitation skills over time; likewise, this study found declines in overall resuscitation knowledge, skills and competency after one year. Large randomized cluster studies are recommended to further determine the effects of training on knowledge, skills and competency retention as well as neonatal morality. This study's finding could prove replicable in similar low-resource settings outside of South Sudan.

### What is known about this topic

- Many studies on HBB demonstrated that training health workers increased knowledge, skills, and competency, and reduced newborn mortality due to asphyxia.

### What this study adds

- HBB training in a tertiary hospital in South Sudan in 2017 increased knowledge, skills and competency. It also contributed to a reduction in early newborn mortality within 24 hours after one year of implementation.

## Competing interests

The authors declare no competing interests.
